# Targeting Nociceptive Neurons and Transient Receptor Potential Channels for the Treatment of Migraine

**DOI:** 10.3390/ijms24097897

**Published:** 2023-04-26

**Authors:** Cinder Faith Cohen, Jueun Roh, Sang Hoon Lee, Chul-Kyu Park, Temugin Berta

**Affiliations:** 1 Pain Research Center, Department of Anesthesiology, Medical Center, University of Cincinnati, Cincinnati, OH 45219, USA; cohencf@mail.uc.edu (C.F.C.); jueun9392@gmail.com (J.R.); lee4s2@ucmail.uc.edu (S.H.L.); 2 Neuroscience Graduate Program, College of Medicine, University of Cincinnati, Cincinnati, OH 45267, USA; 3 Department of Physiology, Gachon Pain Center, College of Medicine, Gachon University, Incheon 21936, Republic of Korea

**Keywords:** migraine, headache, peripheral sensory neurons, transient receptor potential channels, TRPC4, CGRP

## Abstract

Migraine is a neurovascular disorder that affects approximately 12% of the global population. While its exact causes are still being studied, researchers believe that nociceptive neurons in the trigeminal ganglia play a key role in the pain signals of migraine. These nociceptive neurons innervate the intracranial meninges and convey pain signals from the meninges to the thalamus. Targeting nociceptive neurons is considered promising due to their accessibility and distinct molecular profile, which includes the expression of several transient receptor potential (TRP) channels. These channels have been linked to various pain conditions, including migraine. This review discusses the role and mechanisms of nociceptive neurons in migraine, the challenges of current anti-migraine drugs, and the evidence for well-studied and emerging TRP channels, particularly TRPC4, as novel targets for migraine prevention and treatment.

## 1. Introduction

Approximately 12% of the global adult population suffers from migraine [1], a disorder characterized by recurrent, severe, one-sided, or two throbbing headaches that can be accompanied by nausea, vomiting, photophobia, and cutaneous allodynia [2,3]. Although the exact causes of migraine are still being studied and debated, it is generally considered to be a neurovascular disorder with a complex interplay of both neurological and vascular factors.

The vascular component of migraine is indicated by the throbbing nature of the headache; however, this theory remains controversial, with some researchers regarding vascular changes as an epiphenomenon [4]. In contrast, the neurological nature of migraine is better understood, with evidence suggesting that it is influenced by factors such as anxiety, stress, and cortical spreading depression (CSD) [5]. Approximately one-third of those who suffer from migraine also experience an aura, which is a manifestation of transient visual and somatosensory disturbances induced by CSD; a slowly propagating wave of neuronal and glial depolarization across the cortex [6,7,8]. In addition, CSD is believed to activate sensory neurons in the trigeminal ganglia (TG), suggesting the role of the central and peripheral nervous systems in migraine [9,10].

**Figure 1:**
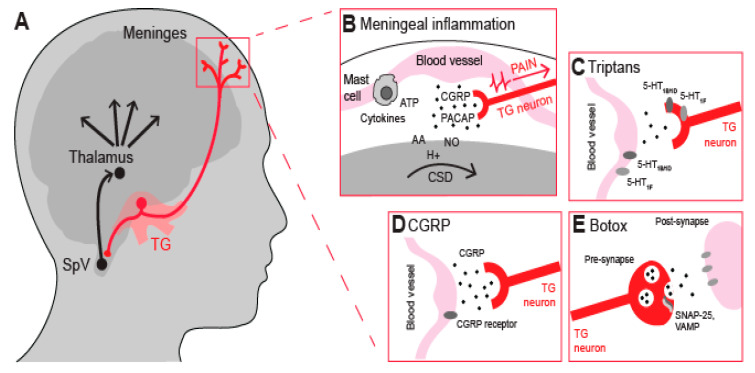
Physiology of the migraine pathogenesis and pharmacological targets. (**A**) Migraine pain axis comprising peripheral sites such as the trigeminal ganglia (TG) and meninges as well as central nervous system structures, including the spinal trigeminal nucleus (SpV) and thalamus. (**B**) Nociceptor activation or cortical spreading depression (CSD) triggers the release of inflammatory and nociceptive neuropeptides from the trigeminal terminals innervating the meninges and associated blood vessels. These neuropeptides induce vasodilation and sterile neurogenic inflammation involving mast cell degranulation and release of pro-inflammatory mediators. This, in turn, leads to the sensitization of second-order neurons in the SpV, which increases nociceptive signaling to the thalamus and higher-order cortical brain structures that process pain. (**C**–**E**) Additionally depicted is the contribution of CGRP to migraine pathophysiology and the mechanism of action of triptans and Botox.

In the peripheral nervous system, nociceptive neurons in the TG are thought to play a key role in the pain signals of migraine [11,12], as they innervate the intracranial meninges and convey pain signals from the meninges to the thalamus (Figure 1A). Targeting these nociceptive neurons is considered a promising approach for migraine treatment owing to their accessibility and distinct molecular profile [13], which includes the expression of several transient receptor potential (TRP) channels that are being investigated as potential therapeutic targets for migraine [14,15,16]. In this review, we discuss the role and mechanisms of nociceptive neurons in migraine, the challenges of current anti-migraine drugs, and the evidence for well-studied and emerging TRP channels as novel targets for migraine prevention and treatment.

## 2. Nociceptive Neurons and Meningeal Inflammation

Sensory neurons located in the dorsal root ganglion (DRG) and TG are responsible for detecting and transmitting sensory information from the periphery to the central nervous system (CNS) [17]. Nociceptive neurons are specialized peripheral sensory neurons that encode painful stimuli [18]. Activation and sensitization of nociceptive neurons innervating cephalic tissues, mainly the cranial meninges and their associated blood vessels, are the basis of migraine pain [12]. Nociceptive neurons can release neuropeptides associated with migraines, such as calcitonin gene-related peptide (CGRP), serotonin, and pituitary adenylate cyclase-activating polypeptide-38 (PACAP-38). This can lead to vasodilation and meningeal inflammation, which can trigger migraine attacks [19,20,21,22].

Meningeal inflammation, triggered by various cells and molecules, can activate and sensitize nociceptive neurons (Figure 1B), leading to migraine attacks [23,24,25,26]. Following CSD, molecules such as ATP, glutamate, K+, H+, arachidonic acid (AA), and nitrous oxide (NO) are released locally and are thought to diffuse towards and activate meningeal nociceptive neurons [27,28,29,30]. This occurs a few minutes after CSD, which is consistent with the time delay between the onset of aura and migraine attack [9]. Previous studies have observed that mast cell degranulation, which involves the release of ATP, histamine, and pro-inflammatory cytokines, can cause meningeal inflammation and long-term activation and sensitization of dural nociceptive neurons [26]. In fact, injections of the mast cell degranulator compound 48/80 in mice resulted in migraine-associated behaviors, some of which were reversed by mast cell stabilizers and anti-inflammatory drugs [31,32].

Targeting nociceptive neurons and meningeal inflammation represents a viable strategy for preventing and relieving migraine pain. Current migraine pharmacotherapies can be divided into two main categories: abortive therapies, which aim to end a migraine episode, and prophylactic therapies, which aim to prevent future migraine attacks. Several classes of drugs are used to prevent migraines, such as anti-epileptics, anti-hypertensive agents, tricyclic antidepressants, anti-CGRP antibodies, and botulinum toxin [27,33,34,35,36]. Abortive treatments mostly include triptans and non-steroidal anti-inflammatory drugs (NSAIDs) [37]. The following paragraphs provide a brief overview of how these therapies affect nociceptive neurons and meningeal inflammation without providing clinical advice or indications.

## 3. Current Anti-Migraine Drugs

NSAIDs are widely available as over-the-counter medications for pain relief and are often used to treat mild to moderate acute migraine [38]. These drugs possess anti-inflammatory, analgesic, and antipyretic properties, mostly by blocking the enzymes cyclooxygenase-1 and -2 (COX-1 and -2) and thus reducing prostaglandin synthesis. It is important to note that COX-1 is widely distributed and involved in homeostatic mechanisms, while COX-2 is mainly expressed in areas of inflammation and is responsible for anti-inflammatory and analgesic effects [39]. NSAIDs with selective inhibition of COX-2 may be considered a relatively safer treatment for migraine, although they are associated with more adverse cardiovascular effects [40].

Triptans targeting 5-HT receptors are a gold-standard treatment for migraine attacks [36]. They block nociceptive signaling to second-order neurons in the trigeminal nucleus caudalis but have limited ability to cross the blood–brain barrier (BBB). Their primary action is in the periphery (Figure 1C). Triptans are 5-HT1B/1D receptor agonists, causing vasoconstriction via 5-HT1B and inhibiting neuropeptide release via 5-HT1D [41]. However, they increase blood pressure and, therefore, may be contraindicated in those with cardiovascular disease or hypertension and pregnant women [42,43]. Another concern with the chronic use of triptans is the development of medication overuse headaches [44]. Ditans are 5-HT1F agonists that do not cause vasoconstriction due to low affinity for the 5-HT1B receptor [41]. Ditans are often used for the treatment of migraine in patients with or at risk of cardiovascular disease and in patients who respond poorly to their current treatment [45].

Despite CGRP being identified as a key molecule in migraine pathology more than 30 years ago, drugs acting on the CGRP or its receptor have only recently been approved for migraine treatment [46]. These drugs can be divided into two main groups: small-molecule CGRP receptor antagonists and monoclonal antibodies targeting either CGRP or its receptor [46,47]. Both small-molecule antagonists and antibodies do not easily cross the BBB; hence, they mainly act peripherally on the trigeminal ganglion and meninges (Figure 1D). Monoclonal antibodies are generally considered safe and effective for migraine prevention and treatment; however, long-term CGRP blockade effects remain unknown and may be a concern for patients with or at risk of cardiovascular diseases [48,49].

Botulinum neurotoxin serotype-A (BoNT/A) is emerging as a potential treatment for chronic migraine [50]. It has been well-documented that BoNT/A interferes with synaptic vesicles, blocking the release of neuropeptides (Figure 1E) [51,52]. For instance, a clinical study has shown that BoNT/A reduces interictal CGRP plasma levels in chronic migraine patients [53]. However, it is becoming increasingly clear that BoNT/A also regulates the expression and function of TRP channels in nociceptive neurons, which may explain its analgesic effect by inhibiting CGRP release [54]. The effects of BoNT/A on TRP channels have not yet been tested directly in migraineurs. However, the reported analgesic response to BoNT/A injections in people injected with capsaicin is likely due to a decrease in expression of the TRP cation channel subfamily V member 1 (TRPV1) in nociceptive neurons [55] and the potential analgesic response to BoNT/A treatments in a population of female patients with chronic migraine may be associated with a particular polymorphism in the TRPV1 gene [56]. The success of BoNT/A and its interactions with TRPV1 provide support for targeting TRP channels in migraine treatment.

## 4. TRP Channels as Targets for Anti-Migraine Drugs

TRP channels represent a non-selective ion channel superfamily grouped into several subtypes based on sequence homology [57]. In mammals, these are classified into six subfamilies: TRPA (ankyrin), TRPV (vanilloid), TRPC (canonical), TRPM (melastatin), TRPML (mucolipin), and TRPP (polycystin) [58]. TRP channel members are structurally similar, with six transmembrane domains (S1–S6) and cytoplasmic amino and carboxy termini. However, they differ in their primary amino acid sequence [59]. The S1–S6 domains assemble into homo- or hetero-tetramers, with a hydrophilic loop between the fifth and sixth transmembranes forming a pore that is permeable to cations such as Na^+^ and Ca^2+^ ions. TRP channels can be triggered by various stimuli, such as thermal, mechanical, chemical, pH, and osmolarity, as well as external and internal ligands [58]. Consequently, they are implicated in many diseases [60]. TRP channels are particularly abundant in sensory neurons and have been linked to various pain conditions [61]. Evidence suggests that they activate meningeal nociceptive neurons, contributing to the development and progression of migraine [14,15]. For instance, TRP channels are sensitive to environmental factors, such as pollutants and temperature changes, which are known migraine triggers [62,63,64]. Additionally, their activation induces transcriptional changes and the release of pro-inflammatory and algesic neuropeptides, such as CGRP and substance P (SP), from TG nociceptive neurons [10]. Moreover, polymorphisms in TRP genes may also regulate the propensity for migraine and responsiveness to its treatment [56,65]. Pre-clinical studies have further supported the role of TRP channels in migraine and have shown that their silencing or inhibition can reduce migraine pain (Table 1).

TRPV1, the first of many thermosensitive TRP channels to be identified, functions as a noxious heat sensor that is activated by temperatures greater than 42 °C and pH changes [87,88]. Additionally, it is triggered by exogenous stimulants, such as capsaicin from chili peppers, and endogenous stimulants, such as anandamide, produced in inflammatory processes [87,88,89]. TRPV1 activity is further modulated by inflammatory mediators, such as prostaglandin E2 and bradykinin, which trigger the release of pro-inflammatory and pro-migraine neuropeptides, including CGRP and SP, within the meninges [90,91]. Therefore, TRPV1 has been thoroughly studied in migraine [92]. TRPV1 is highly expressed in small and medium-sized neurons in the peripheral ganglia, with approximately 10–20% of TG neurons reported to be TRPV1-positive [93]. Additionally, 70% of CGRP-positive neurons have been shown to co-localize with TRPV1-positive neurons in the TG, and dural trigeminal fibers also exhibit co-localization [94,95]. Genetic evidence suggests the involvement of TRPV1 in migraine, as a study conducted in the Spanish population found single nucleotide polymorphisms (SNPs) in the TRPV1 gene in patients with migraine [96]. Furthermore, the anti-migraine drug sumatriptan was recently shown to block trigeminal TRPV1 channels [71]. Thus, TRPV1 agonists and antagonists have been identified as potential therapeutic agents for migraine management [97]. However, several promising pre-clinical drugs directly targeting TRP channels have failed to translate into clinical use or have severe side effects [61]. Stimuli caused by capsaicin are very intense; however, repeated intranasal application of capsaicin can cause nociceptive fibers to become insensitive not only to capsaicin itself but to other stimuli as well. This can lead to a significant reduction in migraine attacks in migraineurs, but adverse side effects, including nasal burning and lacrimation, prevent the clinical application of these drugs [90,98]. Since then, the focus of most TRPV1 drugs for migraine treatment has been on TRPV1 antagonists [90]. Unfortunately, SB-705498, a TRPV1 antagonist, failed to demonstrate superior efficacy against migraine headaches compared to a placebo in Phase II clinical trial [66]. Moreover, other studies testing TRPV1 antagonists were terminated due to serious adverse effects, notably hyperthermia [99]. Adverse side effects of TRP channel targeting may be avoided by indirect inhibition using resolvins, which have no apparent thermoregulatory effects [100]. Notably, we found that resolvin D3 significantly inhibited TRPV1 activity and CGRP release in both mouse and human DRG neurons [101] and may represent a new anti-migraine drug.

TRPA1 is another calcium-permeable non-selective cation channel that was initially thought to detect noxious cold temperatures with a threshold of 17 °C [102,103]. However, later studies indicated that TRPA1 is involved in detecting cold hypersensitivity rather than physiological cold pain [104]. TRPA1 can be activated by a variety of environmental irritants, pollutants, and pungent food such as garlic, cinnamon, and mustard oil [105,106,107]. Additionally, the monoterpene umbellulone, produced by Umbellularia californica, has been linked to migraine induction in humans and reported as a TRPA1 activator [63,108]. In rodents, umbellulone administration has been shown to result in CGRP release, meningeal vasodilation, and migraine-like behaviors [63]. TRPA1 is also linked to migraine through its endogenous activators, such as reactive oxygen species (ROS), reactive prostaglandins, and nitric oxide (NO), all of which are well-known to play a role in migraine attacks [14]. Rodent studies have shown that TRPA1 is mainly localized to small and medium-sized peptidergic neurons and is present in around 36% of trigeminal sensory neurons and dural afferents [10,93]. In addition, it has been found that TRPA1 is present in a subset of C and Aδ neuronal fibers expressing TRPV1, CGRP, SP, and protease-activated receptor 2 (PAR-2) [10,95,109]. Studies have shown that the PAR-2 receptor can induce migraine pain behavior in mice, and a PAR-2 monoclonal antibody has been recently tested in rodents as an anti-migraine drug [31,110]. Pre-clinical studies have also shown that several TRPA1 antagonists and genetic deletion are effective in reducing migraine-like symptoms such as allodynia [72,73,74]. However, it is important to note that allodynia, but not vasodilation, was prevented through the genetic deletion or pharmacological blockade of TRPA1 [73]. Further evidence establishing TRPA1 as a therapeutic target for migraine comes from in vivo and in vitro pre-clinical studies. These studies showed that plant extracts from the feverfew herb (Tanacetum parthenium) and butterbur (Petasites hybridus) desensitize meningeal TRPA1 channels, resulting in decreased release of vasoactive neuropeptides and consequent pain relief [77,78]. Currently, high-affinity and selective TRPA1 antagonists are in the developmental phase and undergoing phase I and phase II clinical trials for different diseases that have a significant pain component [92].

TRPM8 is activated by mild cold temperatures ranging from 8 to 25 °C, as well as by cooling agents such as menthol and icilin [111,112]. TRPM8 can also be activated and regulated by various endogenous molecules, including lipids, artemin, testosterone, and membrane phosphatidylinositol 4,5-bisphosphate (PI(4,5)P2) [113,114,115,116]. Single nucleotide polymorphisms (SNPs) in TRPM8 have been associated with migraine [117,118,119]; however, it is unclear whether some of these SNPs are beneficial or detrimental. For instance, SNP rs10166942 has been associated with a lower risk of migraine, but it has been linked to the development of chronic migraine and allodynia in some patients [117,118,120]. Cold temperatures are also known to trigger migraines, and several migraine patients exhibit cold allodynia [64,121]. Although cold temperature and allodynia may suggest a detrimental role for TRPM8, the TRPM8 agonist menthol offers pain relief in some migraine patients [82]. In mice, TRPM8 is expressed in approximately 12% of potentially peptidergic neurons in the trigeminal ganglion [93]; however, only a small percentage of TRPM8-containing neurons also express CGRP and TRPV1 [122,123,124]. Although it is clear that TRPM8 mediates both innocuous and noxious cold sensations, as well as being necessary for cold allodynia and hyperalgesia in various pain models, the role of this channel in migraine-related pain is controversial [125]. For instance, it has been shown that the activation of meningeal TRPM8 by exogenous agonists can both cause and alleviate headache behaviors, depending on whether other meningeal afferents concurrently receive noxious stimuli [79,80]. A recent study using transgenic knockout TRPM8 mice suggests a protective role in promoting a faster recovery from chronic migraine-like symptoms in male mice compared to that in female mice [126]. However, another study with transgenic mice showed that TRPM8 channels or afferents are both required for the development of acute and chronic migraine-like symptoms [127]. This suggests that further investigations are needed to determine the role and therapeutic potential of TRPM8.

TRPV4 responds to various stimuli, including changes in osmolarity and mechanical forces imposed on the cell membrane, suggesting that it functions as a mechanosensory receptor [128,129]. Although little is known about the relationship between TRPV4 and migraine, its function is particularly intriguing as changes in intracranial pressure are known to affect headaches. TRPV4 is expressed in meningeal nociceptive neurons with PAR2, and activating this channel in the dura causes headache behavioral responses, such as cephalic and extracephalic allodynia [84,130]. A TRPV4 antagonist inhibited these behavioral responses, suggesting that this channel can be a potential therapeutic target for anti-migraine drugs.

## 5. TRPC4 Channel as an Emerging Target for Anti-Migraine Drugs

The TRP channel subfamily known as transient receptor potential canonical (TRPC) consists of seven members, TRPC1–7, which can be further divided into four subgroups: TRPC1, TRPC4/5, TRPC3/6/7, and TRPC2, based on sequence similarity [131]. TRPC channels are expressed across species, with the exception of TRPC2, which is not expressed in humans [132]. These channels possess six transmembrane helices and assemble into cation channels as homo- and hetero-tetramers, including two or three TRPC subtypes [133]. They are permeable non-selective cation channels that are activated by G-protein-coupled receptors or receptor tyrosine kinases. Most of these channels are linked to phospholipase C signaling and calcium signaling [134,135,136,137]. Recent studies have revealed that these channels play an integral role in neuronal functions and brain diseases through their regulation of intracellular Ca^2+^, which contributes to synaptic transmission and neural plasticity [138]. For instance, studies have shown that TRPC4 and TRPC5 are highly expressed in brain regions associated with anxiety and fear, and genetic silencing or pharmacological inhibition of these channels have been found to be effective in pre-clinical animal models of depression [139,140,141]. TRPC4 and TRPC5 are expressed not only in the brain but also in peripheral sensory neurons, where they are involved in axon guidance and sensory functions [85,86,142,143,144].

TRPC4 expression has been initially identified in sensory neurons from dorsal root ganglia as a major driver of serotonergic and psoriasiform itch. First, we found that TRPC4 has a unique function in DRG neurons, mediating itch to serotonergic antidepressants independently of TRPV1 and TRPA1 [145]. Second, we characterized the expression of TRPC4 in peptidergic DRG neurons and showed that acute itch induced by serotonin and histamine was attenuated in TRPC4 knockout mice and mice treated with ML204 [142], a specific TRPC4 antagonist [146,147]. More importantly, pharmacological inhibition of TRPC4 resulted in the reduction of psoriasiform skin inflammation and itch, potentially through a decrease of cutaneous pro-inflammatory cytokine and CGRP levels [142]. This suggests that TRPC4, via its expression in primary sensory neurons and regulation of CGRP, may represent a novel therapeutic target for migraine.

**Figure 2:**
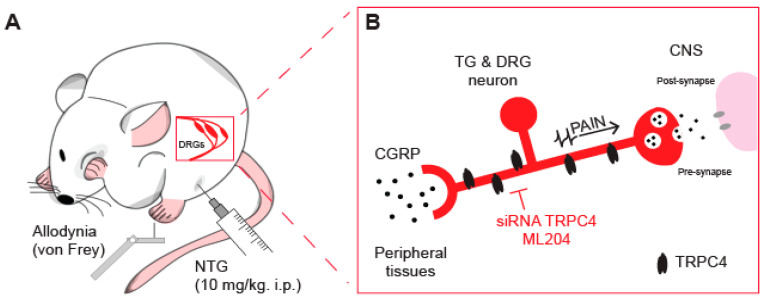
TRPC4 inhibition alleviates nitroglycerin (NTG)-induced migraine in mice. (**A**) Illustration of the mouse models used for acute (episodic) and chronic migraine, which consist of single or repeated intraperitoneal (i.p.) injections of NTG. NTG induces mechanical allodynia that can be assessed by applying von Frey filaments to the hind paw. (**B**) Inhibiting TRPC4 with the ML204 antagonist or small interfering RNA (siRNA) has the potential to exert anti-migraine effects by blocking both CGRP release in peripheral tissues and pain transmission to the central nervous system (CNS).

A recent study showed that TRPC4 is highly expressed in TG neurons and that pharmacological inhibition of TRPC4 significantly prevented migraine-linked cutaneous mechanical hypersensitivity and increased plasma levels of CGRP [86]. Specifically, we found that TRPC4 is highly expressed in TG tissues and neurons, with most of these neurons also expressing CGRP and innervating the skin. To investigate whether TRPC4 plays a role in migraine, we used mouse models of migraine induced by intraperitoneal administration of nitroglycerin (NTG, Figure 2A). A single injection mimicked an acute episodic migraine attack, while repeated injections mimicked chronic migraine [148]. Mice injected with NTG develop cutaneous mechanical hypersensitivity, also known as allodynia, in the periorbital area and hind paw, which is a symptom often reported by migraineurs [149]. We observed that the TRPC4-specific inhibitor ML204 reduced cutaneous mechanical hypersensitivity following a single injection and prevented its development after repeated injections. Interestingly, our results indicate that both male and female mice experienced similar reduction and prevention of mechanical hypersensitivity, despite the fact that female mice may have different underlying mechanisms and women are generally more susceptible to migraines [150,151]. ML204 may be effective in both sexes since it targets multiple pathways associated with inflammatory mediators, including the substance P and serotonin, which are implicated in migraine [22,152]. Additionally, CGRP plasma levels were reduced in both male and female mice after daily treatment with ML204. A recent report suggests that CGRP may have female-specific actions in peripheral tissues [150]; however, as a small-molecule inhibitor, ML204 can act in both peripheral and central nervous system tissues. Thus, ML204 can target the release of CGRP in the brainstem, which is important in migraine pathophysiology. However, no study has evaluated the role of the male vs. female brainstem in migraine [153,154]. Previously, we showed that ML204 and small interfering RNA targeting TRPC4 could attenuate the activity of sensory neurons [145]. This suggests that targeting TRPC4 can lead to analgesia by reducing neural activity and the release of CGRP (Figure 2B). Since TRPC4 is expressed in human DRG tissue [145] and TRPC4/5 has entered clinical trials for the treatment of central nervous system disorders, it is possible that TRPC4 antagonists may soon be useful in migraine treatment.

## 6. Conclusions

Migraine is a complex disorder with both neurological and vascular components. The exact causes of migraine are still being studied and debated. It is thought that nociceptive neurons in the trigeminal ganglia play a key role in the pain signals of migraine, as they innervate the intracranial meninges and convey pain signals from the meninges to the thalamus. Because these nociceptive neurons are accessible and have a distinct molecular profile, targeting them is considered a promising approach. Among the TRP channels, TRPV1, TRPA1, and TRPM8 have been well studied and are being investigated as potential therapeutic targets for migraine prevention and treatment. Apart from these, TRPC4 is another subtype of TRP channels that have been implicated in pain and migraine. We found that TRPC4 is highly expressed in TG neurons and that inhibiting TRPC4 can prevent migraine-linked cutaneous mechanical hypersensitivity and reduce CGRP plasma levels in a mouse model of migraine. Thus, targeting TRP channels, including TRPC4, represents a promising approach for the prevention and treatment of migraine, a debilitating neurological disorder that affects millions of people worldwide.

## Figures and Tables

**Table 1:** Studies exploring the role of TRP channels in migraine.

	Model Tested	Approach	Effect	Reference
TRPV1	Capsaicin injection in the carotid artery–rats	TRPV1 antagonists: JNJ-38893777, JNJ-17203212	↓ CGRP	[66]
	Dural application of TRPV1 agonist capsaicin–rats	Botulinum toxin A Injections	↓ response of C-type meningeal nociceptive neurons	[67]
	Electrode stimulation of the dura above the middle meningeal artery–rats	TRPV1 agonist: olvanil	↓ activity of the trigeminocervical complex	[68]
	EtOH-treated TG tissue slices–guinea pigs	TRPV1 antagonist: capsazepine	↓CGRP & SP	[69]
	Meningeal application of TRPV1 agonist capsaicin-rats	TRPV1 antagonist: capsazepine	↓ activity TG fibers	[70]
	Capsaicin-treated TG neurons and trigeminal nucleus caudalis–rats	Sumatriptan	↓ TRPV1 current ↓ sEPSC frequency	[71]
TRPA1	Systemic NTG administration and orofacial formalin test–rats	TRPA1 antagonist–ADM_12	↓ nocifensive behavior	[72]
	Systemic Glyceryl trinitrate administration-mice	TRPA1 antagonist–HC-030031, TRPA1-conditional KO mice	↓ mechanical allodynia and activity of TG neurons	[73]
	Systemic NTG administration–mice	TRPA1 antagonist–HC-030031	↓ mechanical allodynia	[74]
	Dural application of TRPA1 agonists: mustard oil or umbellulone-rats	TRPA1 antagonist–HC-030031	↓ mechanical allodynia, ↑ rearing in Open Field Test	[75]
	Mouse brain slice cortical spreading depression (CSD) model treated with TRPA1 agonist–Umbellulone	TRPA1 antagonists: HC-030031, A967079, anti-TRPA1 antibody	↓ CSD	[76]
	Dural application of TRPA1 agonist-allyl isothiocyanate	TRPA1 partial agonist–parthenolide	↓ mechanical allodynia	[77]
	Facial or bath (culture) application of TRPA1 agonist-allyl isothiocyanate	TRPA1 agonist–isopetasin	↓ nocifensive behavior and activity of TG neurons	[78]
TRPM8	Dural application of icilin–rats	TRPM8 antagonist–AMG-1161	↓ mechanical allodynia	[79]
	Dural application of inflammatory mediators–mouse	TRPM8 agonist–menthol	↓ nocifensive behavior	[80]
	Dural application of inflammatory soup-mice	TRPM8 agonist–icilin	↑ heat threshold temperature	[81]
	Cutaneous application of menthol–human	TRPM8 agonist–menthol	↓ migraine	[82]
TRPV4	Formalin injections to whiskerpad-rats	TRPV4-KO mice and TRPV4 antagonist–HC067047	↓ nocifensive behavior	[83]
	Dural application of hypotonic solution TRPV4 agonist–4α-PDD–rats	TRPV4 antagonist -RN1734	↓ nocifensive behavior	[84]
TRPC4/5	Dural injections of lysophosphatidylcholine-mice	TRPC5 antagonist–AC1903	↓ mechanical allodynia	[85]
	Systemic NTG administration-mice	TRPC4 antagonist-ML204	↓ mechanical allodynia; ↓ CGRP plasma levels	[86]

## Data Availability

Not applicable.
